# Acute appendicitis in pediatric patients with Coronavirus Disease 2019 (COVID-19): A case series from a developing country's tertiary hospital

**DOI:** 10.1016/j.amsu.2022.103315

**Published:** 2022-01-26

**Authors:** Nurnaningsih, Gladys Indika Danudibroto, Desy Rusmawatiningtyas, Intan Fatah Kumara, Firdian Makrufardi, Titis Widowati

**Affiliations:** a Department of Child Health, Faculty of Medicine, Public Health and Nursing, Universitas Gadjah Mada/Dr. Sardjito Hospital, Yogyakarta, 55281, Indonesia; b Department of Radiology, Faculty of Medicine, Public Health and Nursing, Universitas Gadjah Mada/Dr. Sardjito Hospital, Yogyakarta, 55281, Indonesia

**Keywords:** COVID-19, Appendicitis, Pediatrics, Case series

## Abstract

**Introduction:**

and importance: A common gastrointestinal presentation of both COVID-19 and multisystem inflammatory syndrome in children (MIS-C) is acute abdominal pain, which sometimes mimics appendicitis. Literature describing children with COVID-19 infection and concurrent acute appendicitis is growing, and understanding these patients’ clinical picture is necessary for their proper treatment.

**Case presentation:**

We present a case series of six healthy children before they developed classic symptoms of appendicitis. At the same time, they were also found to have confirmed COVID-19. All patients had fever and right lower abdominal pain. Four of six children having Alvarado score above seven had surgical treatment, while the others only received systemic antibiotic and antiviral medication. Surgical results of two patients revealed perforated appendicitis. No mortality occurred among them.

**Clinical discussion:**

There is increasing recognition of gastrointestinal involvement in patients with COVID-19 and MIS-C. There are several postulates to explain appendicitis in COVID-19. First, inflammatory response is exaggerated in SARS-CoV-2 infected patients. Second, obstruction of the appendiceal lumen is caused by mesenteric adenopathy, which in turn, is caused by COVID-19 infection, not fecalith. Third, hyperinflammatory response in MIS-C triggers inflammation in appendix.

**Conclusion:**

Clinicians must recognize that abdominal pain with fever could be the presenting symptoms of COVID-19 with MIS-C. MIS-C, which has severe presentations with gastrointestinal manifestations and high mortality rate, should be considered as a differential diagnosis for a patient with appendicitis-like symptoms and a positive SARS-CoV-2 infection.

## Highlights

• Inflammatory response is exaggerated in SARS-CoV-2 infected patients.
• Obstruction of the appendiceal lumen is caused by mesenteric adenopathy.
• Hyperinflammatory response in MIS-C triggers inflammation in appendix.

## 1 Introduction

On December 2020, the outbreak of SARS-CoV-2 infection began in Wuhan, China and spread rapidly all over the world. Currently, several studies postulated that COVID-19 in children appears to be milder in clinical manifestations and the pediatric patients have a lower proportion of symptomatic infection than adults [1]. Children infected with the novel Coronavirus 2019 (COVID-19) may present with a myriad of symptoms, including fever, cough, anosmia, nausea/vomiting, diarrhea, and more [2].

In early May 2020, an increasing amount of evidence emerged in the United Kingdom (UK), the United States, and Europe regarding different manifestations of COVID-19 in pediatric patients, which are nowadays known as the multisystem inflammatory syndrome in children (MIS-C) [3]. Organ (cardiovascular, gastrointestinal, renal, hematology, dermatology, and neurology) involvement of two or more should be included as one of the MIS-C criteria [4]. Eighty-four percent of patients with MIS-C have gastrointestinal symptoms (abdominal pain, nausea, vomiting, diarrhea) as a prominent presenting characteristic [5]. Therefore, our focus was on children who tested positive for COVID-19 with primary symptoms of fever and abdominal pain, as well as the struggle in discerning whether it is a case of appendicitis or a manifestation of MIS-C. This research work has been reported in line with PROCESS criteria [6].

## 2 Case presentation

There were 392 pediatric patients admitted to a tertiary hospital with suspected COVID-19, with 159 of them being tested positive for COVID-19 using SARS COV-2 PCR during COVID-19 pandemic. The study was located in one of the main university-based referral hospitals in Indonesia. We receive pediatric patients from primary and secondary hospitals in Yogyakarta and Central Java region. Among them, six cases of acute appendicitis were reported among nine referral cases of COVID-19 with appendicitis suspicion (Table 1). Patients were 3–15 years old, with four of them being male. All patients presented primary symptoms of both right lower abdominal pain and fever, while four patients had vomiting, and only one patient had diarrhea. All patients were previously healthy children, with two of them being obese. Initial assessment at admission showed two patients were in shock and were obese, which posed comorbidities, while the others were in a stable condition. Three patients were admitted to intensive care due to MIS-C.

**Table 1:** Characteristics of research subjects.

Characteristics	Patient 1	Patient 2	Patient 3	Patient 4	Patient 5	Patient 6
Age (years)	3	11	15	7	9	5
Gender	Male	Male	Female	Female	Male	Male
Comorbidity	Obesity	None	None	None	Obesity	None
Presenting symptoms	7 days of generalized abdominal pain, vomiting, fever	2 days of generalized abdominal pain, fever	2 days of generalized abdominal pain, vomiting, fever	2 days of generalized abdominal pain, vomiting, fever and diarrhea	6 days of generalized abdominal pain, vomiting, fever	5 days of generalized abdominal pain, fever
Initial assessment	In shock	stable condition	stable condition	stable condition	In shock	stable condition
Alvarado score	9	8	10	10	6	4
**Initial lab**						
Hemoglobin (g/dL)	10 (normal)	12.3 (normal)	14.4 (normal)	15.2 (normal)	11.4 (normal)	11.2 (normal)
White blood count (x10^3^/μL)	25.92 (↑)	17.07 (↑)	15.05 (↑)	19.14 (↑)	26.67 (↑)	19.02 (↑)
Neutrophils (x10^3^/μL)	95 (↑)	79 (↑)	91.3 (↑)	87.9 (↑)	79.5 (↑)	89.4 (↑)
Lymphocytes (x10^3^/μL)	2.2 (↓)	10.4 (↓)	5 (↓)	6.6 (↓)	8.1 (↓)	7.2 (↓)
Platelets (x10^3^/μL)	217 (normal)	316 (normal)	206 (normal)	352 (normal)	636 (normal)	355 (normal)
Blood urea nitrogen (mg/dL)	18.0 (normal)	11 (normal)	12.2 (normal)	14.2 (normal)	44.6 (↑)	Not performed
Creatinine (mg/dL)	0.81 (normal)	0.55 (normal)	0.85 (normal)	0.67 (normal)	6.73 (↑)	Not performed
Aspartate transaminase (U/L)	7 (normal)	15 (normal)	18 (normal)	21 (normal)	29 (↑)	Not performed
Alanine transaminase (U/L)	11 (normal)	11 (normal)	22 (normal)	14 (normal)	78 (↑)	Not performed
CRP	>150 (↑)	>150 (↑)	>150 (↑)	>150 (↑)	31 (↑)	Not performed
Procalsitonin	10.86 (↑)	0.9 (↑)	0.7 (↑)	52.94 (↑)	31.5 (↑)	Not performed
IL-6	26.71 (↑)	17.39 (↑)	84.61 (↑)	322.2 (↑)	191.5 (↑)	Not performed
Respiratory problems	No	No	No	No	No	No
MIS-C	Yes	No	No	Yes	Yes	No
Thorax x-ray	Covid-19 bilateral pneumonia	Within normal limits	Covid-19 bilateral pneumonia	Covid-19 bilateral pneumonia	Covid-19 bilateral pneumonia	Within normal limits
Culture	no bacterial growth	no bacterial growth	Not performed	*Escherichia coli*	*Klebsiella pneumoniae*	Not performed
Pathology findings	acute exacerbation of chronic appendicitis	appendicitis and chronic periappendicular suppuration	appendicitis and acute periappendicular suppuration with perforation	Appendicitis and periappendicular suppuration with perforation	Not performed	Not performed
Antibiotics	Yes	Yes	Yes	Yes	Yes	Yes
Antiviral	Yes	No	Yes	Yes	Yes	No
Intensive care	Yes	No	No	Yes	Yes	No
Length of stay (days)	14	7	9	8	16	5
Mortality	No	No	No	No	No	No

Hemoglobin levels were found normal, while levels of leucocyte, neutrophil, CRP, procalcitonin were elevated in all patients. All patients had tested positive for SARS-CoV-2, but none of them developed respiratory symptoms, and three of them had MIS-C. One of the three patients with MIS-C had increased levels of both aspartate and alanine transaminase. Positive culture was found in two patients; one result was from peritoneal fluid (*Escherichia coli*), and the other was from feces (*Klebsiella pneumoniae*). All patients had sterile blood cultures. Two patients, who showed normal chest X-ray results, were either asymptomatic or had a mild degree of COVID-19 infection. The others had COVID-19 bilateral pneumonia with moderate to severe degree of COVID-19 infection.

**Fig. 1:**
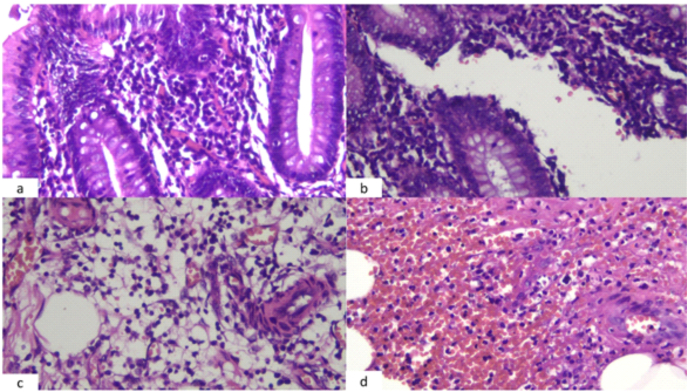
Histopathology finding of appendicitis (a) patient 1; (b) patient 2; (c) patient 3; (d) patient 4.

Four of the six patients with Alvarado score of 7–11 had operative treatment, and appendicitis was confirmed with a pathology examination (Fig. 1.). The two patients with Alvarado score of 4 and 6 underwent conservative treatment with antibiotics and frequent reassessment. From the four operative cases, surgery revealed perforated appendicitis in two patients. In the patients of conservative treatment, abdominal pain disappeared in three and seven days after the start of antibiotic administration. COVID-19's specific medication was not indicated for either asymptomatic or mild degree of COVID-19 infection, while one was for both moderate and severe degree of COVID-19 infection. One patient had renal impairment due to MIS-C and required four times hemodialyses during hospitalization. Length of stay of the patients was 5–16 days. The shortest hospitalization was found in asymptomatic COVID-19 infection, while the longest hospitalization was found in the male patient with obesity risk, in shock, and with MIS-C. Fortunately, all patients have recovered and been discharged.

## 3 Discussion

As of September 30, 2021, nearly 5.9 million children all over the world have tested positive for COVID-19 since the onset of the pandemic [20]. COVID-19 can manifest in different organs, such as respiratory, gastrointestinal, neurology, and genitourinary. Thirty-two percent of the patients had Gastrointestinal (GI) symptoms, such as diarrhea and abdominal pain [7]. Many studies reported an increasing recognition of gastrointestinal involvement in patients with COVID-19 and MIS-C. Available case reports describe patients who had both typical symptoms of appendicitis and positive SARS-CoV-2 PCR or meet the diagnosis for MIS-C [8]. A case found at a tertiary hospital in Jakarta, Indonesia had similar finding with a diagnosis of acute appendicitis with generalized peritonitis [21]. In our study, all of the six patients were diagnosed as confirmed COVID-19, with three of the cases being MISC. All patients were hospitalized due to both fever and right lower abdominal pain as primary concerns.

Since May 2020, there has been an increasing amount of pediatric patients with COVID-19 infection or history of COVID-19 admitted with hyperinflammatory shock and multi-organ involvement, recently known as MIS-C [3]. In May 2020, in the UK, followed by in Italy and New York, the evidence of a different manifestation of COVID-19 in the Centers for Disease Control and Prevention (CDC) was described, which defined the criteria for a reportable case of MIS-C as an individual under the age of 21 years. These criteria include a minimum of 24-h subjective or objective fever ≥38 °C or higher; severe illness necessitating hospitalization; involvement of two or more organ systems; lab evidence of inflammation, elevated levels in at least one of C-reactive protein (CRP), erthyrocyte sedimentation rate (ESR), procalcitonin, fibrinogen, D-dimer, ferritin, lactate dehydrogenase (LDH), interleukin-6 (IL-6), neutrophils, or low albumin; and either positive SARS-CoV-2 testing by reverse transcriptase-polymerase [9]. Patients with MIS-C have both a severe and acute clinical spectrum with higher mortality rate [10]. In patients with MIS-C, gastrointestinal symptoms were found to be 70% [11]. This could overlap with signs and symptoms of acute appendicitis. In a previous study, it has been reported that 11.8% of patients with MIS-C have symptoms mimicking those of appendicitis [12]. Three of our patients met the MIS-C criteria, with two of them being admitted both in shock and in a state of being obese. Obesity is a high risk factor in MIS-C since there is accumulation of inflammatory cells in adipose tissue, and fat tissue-associated cytokines are proinflammatory, which impairs respiratory function as adipose cells have more receptors available for binding to SARS-CoV-2 [13].

Since 70% of MIS-C patients had gastrointestinal symptoms, MIS-C shall be the first priority in differential diagnosis in patients with both gastrointestinal symptoms and a history of recent SARS-COV-2 exposure or infection. Malhotra et al. (2021) identified that children with COVID-19 may present with clinical features suggestive of appendicitis. However, recent studies related to appendicitis and COVID-19 or MISC are still lacking in numbers. There are several postulates regarding appendicitis in COVID-19. First, in COVID-19 patients, the inflammatory response is exaggerated. ACE-2 receptors, a receptor for COVID-19, are widely distributed in both smooth muscle and endothelial cells of the small intestines and colon, as well as arterial and venous endothelial cells throughout the body. Second, obstruction of the appendiceal lumen is caused by mesenteric adenopathy, which in turn is caused by COVID-19 infection, not fecalith. Third, hyperinflammatory response in MIS-C triggers inflammation in appendix [12]. No fecalith was detected in four patients who had surgery, which led us to conclude that these cases of acute appendicitis were caused by COVID-19 infection. Our results are similar with those of Belhadjer et al. who reported a series of cases of urgent abdominal surgery, with all patients having mesenteric lymphadenitis. All of them, who were initially assessed to be in shock and in intensive care management, were patients with MIS-C. In line with our cases, a previous study reported 71% of patients with MIS-C were admitted to the intensive care unit [14].

Appendicitis in our patients were diagnosed based on their medical history, as well as physical and laboratory examination. All of those were applied to the Pediatric Alvarado score. Snyder et al. reported the probability of appendicitis was 80%, 20%, and 6% in patients whose scores were ≥8, 4–7, and <4 [15]. In our series, two of them, who did not receive surgery, scored 6 and 4 in Alvarado score. Conservative management with antibiotics was successful in managing non-perforated appendicitis with strict inclusion criteria [19]. Earlier study postulated the success rate of conservative treatment for non-perforated appendicitis was 73% at 1 year [16]. A case series from South Africa identified four children with appendicitis, confirmed by surgical findings, in the setting of SARS-CoV-2-positive PCR. Meanwhile, MIS-C was found in three of them after operative treatment [17,22]. A systematic review and meta-analysis study showed that up to 41% from 1484 patients was detected SARS-CoV-2 RNA shedding in stool from patients with gastrointestinal symptoms [23]. Another case series identified appendicitis concomitant with acute SARS-CoV-2 infection; all of them did not require surgery and no mortality was found [18].

The limitation of this study is that there was no confirming pathological examination in patients treated with conservative management. This study describes the presentation of cases found in one tertiary hospital, the description of cases in other hospitals and regions may show different results. A further multicenter study is needed to compare our findings.

## 4 Conclusions

Clinician must recognize that abdominal pain with fever could be the presenting symptoms of either COVID-19 or MIS-C. This case series emphasizes that further investigation of COVID-19 and MIS-C should be performed in patients with acute appendicitis. MIS-C, which can have severe manifestations with gastrointestinal involvement and high mortality rate, should be considered as a differential diagnosis for a patient with appendicitis-like symptoms with a positive SARS-CoV-2 infection. Reporting of these case series will help further our understanding of COVID-19 manifestations in children. Further research should be conducted to confirm these findings.

## Ethical approval

The informed consent form was declared that patient data or samples will be used for educational or research purposes. Our institutional review board also do not provide an ethical approval in the form of case report.

## Funding source

This research did not receive any specific grant from funding agencies in the public, commercial, or not-for-profit sectors.

## Author contribution

Nurnaningsih, Gladys Indika Danudibroto, Desy Rusmawatiningtyas, Intan Fatah Kumara, Titis Widowati conceived the study and approved the final draft. Nurnaningsih, Desy Rusmawatiningtyas, Firdian Makrufardi, Intan Fatah Kumara and Titis Widowati drafted the manuscript, and critically revised the manuscript for important intellectual content. Nurnaningsih, Gladys Indika Danudibroto, Desy Rusmawatiningtyas, Intan Fatah Kumara, Firdian Makrufardi and Titis Widowati facilitated all project-related tasks.

## Consent

Written informed consent was obtained from the patient's parents for publication of this case report and accompanying images. A copy of the written consent forms is available for review by the Editor-in-Chief of this journal on request.

## Registration of research studies

researchregistry7479.

## Guarantor

Nurnaningsih.

## Provenance and peer review

Not commissioned, externally peer reviewed.

## Declaration of competing interest

No potential conflict of interest relevant to this article was reported.
